# Nanosensors for Visual Detection of Glucose in Biofluids: Are We Ready for Instrument-Free Home-Testing?

**DOI:** 10.3390/ma14081978

**Published:** 2021-04-15

**Authors:** Luca Boselli, Tania Pomili, Paolo Donati, Pier P. Pompa

**Affiliations:** 1 Nanobiointeractions and Nanodiagnostics, Italian Institute of Technology (IIT), Via Morego 30, 16163 Genova, Italy; tania.pomili@iit.it (T.P.); paolo.donati@iit.it (P.D.); 2 Department of Chemistry and Industrial Chemistry, University of Genova, Via Dodecaneso 31, 16146 Genova, Italy

**Keywords:** nanosensors, glucose, colorimetric test, instrument-free, POC, home testing

## Abstract

Making frequent large-scale screenings for several diseases economically affordable would represent a real breakthrough in healthcare. One of the most promising routes to pursue such an objective is developing rapid, non-invasive, and cost-effective home-testing devices. As a first step toward a diagnostic revolution, glycemia self-monitoring represents a solid base to start exploring new diagnostic strategies. Glucose self-monitoring is improving people’s life quality in recent years; however, current approaches still present vast room for improvement. In most cases, they still involve invasive sampling processes (i.e., finger-prick), quite discomforting for frequent measurements, or implantable devices which are costly and commonly dedicated to selected chronic patients, thus precluding large-scale monitoring. Thanks to their unique physicochemical properties, nanoparticles hold great promises for the development of rapid colorimetric devices. Here, we overview and analyze the main instrument-free nanosensing strategies reported so far for glucose detection, highlighting their advantages/disadvantages in view of their implementation as cost-effective rapid home-testing devices, including the potential use of alternative non-invasive biofluids as samples sources.

## 1. Introduction

Early diagnostics and preventive medicine are increasingly recognized as essential tools to improve public healthcare, yet few clinical solutions are currently available for rapid biomarker analysis due to technological limitations in achieving reliable sensors for frequent screenings. Nevertheless, point-of-care (POC) devices and particularly home-testing kits have already shown their tremendous potential on public health, improving patients’ survival rate and people’s life quality [1,2]. In this framework, the glucometer is probably the most popular home-testing device.

Diabetes mellitus is a chronic, metabolic disease characterized by elevated levels of blood glucose and, according to the World Health Organization, it affects about 422 million people worldwide. Millions of diabetic patients already rely on self-monitoring of blood glucose [3], but the current approaches still present vast room for improvements.

During the 1970s, the idea of a diabetic patient acquiring blood glucose data at home was contemplated; Anton Clemens developed the first blood glucometer combining dry chemistry test strips (Dextrostix) with reflectance photometry. During the 1980s the Dextrometer (Dextrostix with a digital display) was launched, paving the way for the extensive development of new POCs, and blood glucose monitoring became a standard [4]. During the last 40 years, new electrochemical sensors raised, improving sensitivity, accuracy, and precision, and reducing the amount of sample required (to a blood drop), and more recently, continuous glucose monitoring devices were developed [5].

However, most of the commercial glucometers still involve invasive sampling processes, which are quite discomforting for frequent measurements (i.e., venipuncture or finger-prick) or implantable devices that are not cost-effective [6,7] and need to be dedicated to selected chronic patients, thus precluding large-scale monitoring of globally increasing diabetic disorders.

To reduce sampling-related discomfort and healthcare costs (while enlarging the screening scale), the development of instrument-free rapid diagnostic devices is a fundamental step for a real breakthrough in the diagnostic field.

The monitoring of health-related biomarkers by simple colorimetric in vitro tests or paper-based devices (such as lateral flow devices or dipstick systems) [8,9,10,11], significantly cost-effective and flexible, could become a revolutionary screening tool for all people reducing the incidence of chronic pathologies.

The main advantage is that the test results do not need ad hoc reading systems, but they can be visualized by naked-eye (or with a smartphone), requiring no technical skills, and presenting the possibility of testing anywhere at any time with no need of medical personnel.

This article reviews the state-of-the-art of instrument-free glucose detection with a particular focus on nanosensors and non-invasive biofluids. We describe different kinds of colorimetric sensing strategies present in the literature, including those attempting visual glucose detection in biological fluids alternative to blood. Interestingly, some of the approaches and methodologies here summarized can be potentially applied, with minimal changes, to several other biomarkers.

## 2. Biofluids for Glucose Testing

**Figure 1:**
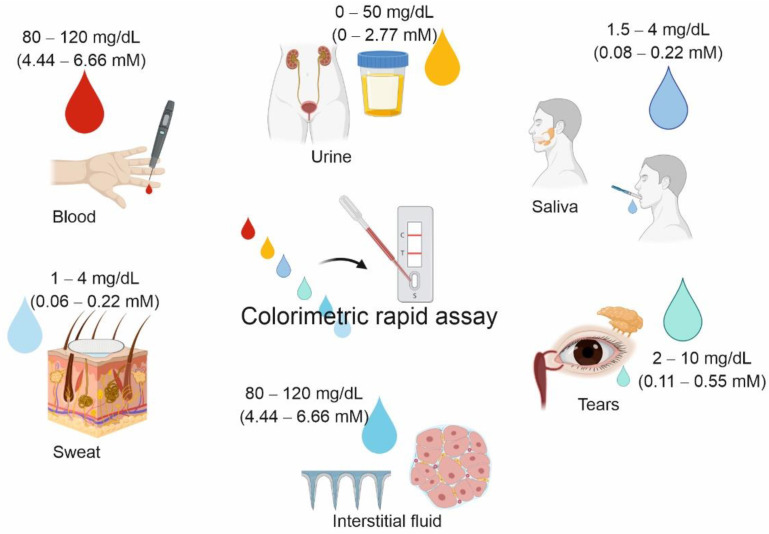
Scheme showing different biological fluids under investigation for their potential as glucose biosources in the development of home-testing systems and their relative physiological glucose concentration.

Blood represents the most investigated biomarker source and the most employed biofluid for diagnostics, followed by urine, while saliva is only recently rising significant interest, e.g., in relation to rapid diagnostic tests for COVID19 [12]. In addition to the biofluids mentioned above, tears, sweat, and interstitial fluid (ISF) can also be employed as biomarker sources. The glucose concentration in the different biofluids can be very different, as reported in Figure 1, in some cases almost two orders of magnitude lower than in blood, requiring technological advancement to obtain the necessary sensitivity. Glucose detection has already been attempted in different biofluids [13,14], and in this review, we summarize the main results obtained for colorimetric nanosensors.

Despite the higher glucose concentration, compared to the other biofluids, blood samples present fast coagulation, intense red color, and a very high concentration of biomolecules, leading to a higher possibility of interference-related problems or limited specificity. Therefore, in most cases, in vitro analyses are performed on serum samples, often diluted even up to 100 times.

Similar to blood, urine, saliva, tears, sweat, and interstitial fluids contain a multitude of constituents (including proteins and metabolites) that reflect biological functions and are currently attracting increasing interest as non-invasive alternative biosources for rapid diagnostics [15,16,17,18,19].

Non-invasive biofluids indeed present several advantages, including needle-less sampling, reduced physical discomfort and anxiety (especially when frequent measurements are needed), no need for trained personnel, and consequently reduced healthcare costs, no risks related to bloodborne pathogens (safer approach), no rapid coagulation (higher stability).

Furthermore, the simplicity of urine and saliva sampling (even for a relatively large amount) allows for specimen self-collection, ideal for home-testing.

Glucose is typically not present in urine (or in very little concentration). However, in the case of elevated levels in the blood, its excretion into urine occurs. Commonly, when glucose concentration is above 50–100 mg/dL (2.77–5.55 mM), the urine test is considered positive for hyperglycemia [20].

Salivary glucose concentration and its correlation with the blood one have been extensively investigated. Depending on the enrolled populations, the exclusion criteria, collection and analysis methods, different correlations have been found for salivary glucose concentration (from 10 to 100 times lower than the hematic one); however, to the best of our knowledge, the most accredited physiological glucose concentration is 1.5–4 mg/dL (0.08–0.22 mM), while values above 4–6 mg/dL (0.22–0.33 mM) represent pathological conditions [21].

Several studies described a relationship between blood and tear glucose levels, and an increase in tear glucose level in diabetic patients was observed after oral glucose load, while no significant rise was found in healthy subjects [22,23,24]. Despite some discrepancies in the correlation between blood and tear glucose, potentially caused by the sample collection methods, tear glucose concentration is commonly reported to be about 2–10 mg/dL (0.11–0.55 mM) [22].

In recent years, thanks to the improvement in sampling techniques for the extraction of biological fluids by absorbent patches and microneedles, sweat and ISF are also emerging as promising alternatives for glucose detection. Sweat glucose has been reported to correlate well with blood level, with a concentration in the 1–4 mg/dL (0.06–0.22 mM) range [25], while blood and ISF show comparable levels in the case of stable values [26]. Nevertheless, tears, sweat, and interstitial fluid present very small sample volumes (hard to collect), which may be particularly suitable for measurement performed by wearable devices [27,28,29,30].

To cope with the low glucose concentration in non-invasive fluids and the ultrahigh sensitivity needed for rapid naked-eye detection, the use of nanoparticles (NPs) represents a great asset to the development of cost-effective kits.

## 3. Nanoparticles as Sensing Tool for Colorimetric Tests

Nanoparticles (NPs), in particular noble metal NPs, hold enormous potential for a new generation of highly sensitive instrument-free colorimetric biosensors [31,32,33,34,35]. Plasmonic NPs, such as AuNPs and AgNPs, thanks to their unique optical properties based on the localized surface plasmon resonance (LSPR), represent a flexible tool for the realization of naked-eye detection for both in vitro testing and paper-based devices like Lateral Flow Devices (LFDs) [36,37,38]. Indeed, these NPs present a high extinction coefficient; their LSPR bands are strictly dependent on the size and shape of the nanostructures and can be extremely sensitive to variations of the chemical environment. Therefore, NP morphological changes lead to spectral variations and color changes. All these aspects opened up a plethora of possibilities for the development of different detection strategies. Common colorimetric approaches involve biomarker-mediated NP aggregation/separation or growth/etching processes [39,40,41,42]. When involving anisotropic shapes, multiple color changes can be obtained, which is particularly advantageous for visual detection as the human eye is more sensitive to light changes in frequency than in intensity [39].

Clearly, given the LSPR sensitivity to the dispersion state and shape variation in in vitro colorimetric tests, the chemical and colloidal stability of plasmonic NPs is of utmost importance.

Recently, various metallic NPs (i.e., Pt, Pd, Au, Fe_3_O, and others) and carbon-based NPs (i.e., graphene oxide and carbon nanotubes) have also emerged as promising candidates to substitute the natural enzymes commonly used in the diagnostic assay [43,44,45,46,47]. Compared to biological enzymes, these so-called *nanozymes* present several advantages, including easy and cost-effective production and purification, stability, resistance to proteases, and high catalytic activity even at extreme pH. Thanks to their peroxidase-like activity, they can be easily coupled with chromogenic substrates typically employed in colorimetric and optical biosensing, such as 3,3′,5,5′-tetramethylbenzidine (TMB), o-Phenylenediamine (OPD), 2,2′-Azinobis [3-ethylbenzothiazoline-6-sulfonic acid (ABTS) and others [44]. Interestingly, bioconjugated nanozymes have been recently employed in paper-based diagnostic devices, e.g., in LFDs, and, in tandem with chromogenic substrates, can lead to highly sensitive colorimetric devices [48].

It is worth mentioning that the molar extinction coefficient of AuNPs is usually (depending on the NP size) up to five orders of magnitude larger than the one of organic chromogens typically employed in colorimetric sensors (i.e., TMB) [49,50]. On the other hand, coupling nanozymes with these chromogenic substrates often lead to very accurate measurements, presenting high reproducibility thanks to enzymatic amplification.

The widespread use of smartphones is promoting the development of portable assays, in which there is no need for specific instrumental readers. Indeed, using smartphone apps as image readers, it is possible to acquire semi-quantitative data from colorimetric sensors [45] and, in some cases, also from light-emitting biosensors (if in the visible range) [51].

For this reason, luminescent NPs such as semiconductor quantum dots, carbon/graphene dots, and polymeric NPs have also been recently exploited to develop rapid, portable sensors by using them merely as reporters (i.e., in LFD-like systems) or involving luminescence color changes and quenching/enhancing strategy (in vitro or on paper-based devices) [37,51,52,53]. For example, a colorimetric paper-based device for visual detection can be developed employing fluorescent paper strips based on dual emission (blue-red) carbon quantum dots (CQDs) [53]. When the paper strip is immersed into a solution containing the targeted analyte, the blue fluorescence is quenched, unlike the red one, leading to a color change.

## 4. Glucose Nanosensors in Different Biofluids

In the following sections, nanosensors for glucose detection in blood, urine, saliva, tears, and sweat will be discussed. To the best of our knowledge, there are currently no examples reported to date for glucose colorimetric nanosensors in ISF.

### 4.1. Blood Glucose Nanosensors

Although its higher glucose concentration compared to the other biofluids (see Figure 1), blood remains a hard sample to handle, even in a laboratory, due to the fast coagulation, the high protein (and metabolites) concentration, and the large presence of cells that are responsible for the characteristic red color. All these factors make whole blood a complex media to work with when aiming at a colorimetric readout, especially for home-testing strategies. For this reason, before laboratory analysis, blood commonly undergoes separation processes and treatments in order to obtain either plasma (cells-depleted blood treated with anticoagulants) or serum (the liquid that remains after the blood clotted).

Several very promising electrochemical approaches, including NPs-based methods, have been proposed for glucose detection; in this context, NPs are commonly employed as electrode materials exploiting their catalytic properties, conductivity, and high surface area (to improve current limits of traditional electrodes) [5,54,55,56,57]. However, this approach requires equipment/reading systems.

This paragraph gives an overview of instrument-free colorimetric nanosensors for glucose detection demonstrated to perform in blood, plasma, or serum samples.

For colorimetric home-testing applications, quick blood purification processes cannot be readily performed unless relying on paper-based devices that involve membranes able to separate the sample components in minutes. Therefore, we look at glucose detection in serum from the perspective of potential future technological translation of the methodology to portable devices.

Glucose can be detected in diluted human serum by H_2_O_2_-induced sol-gel transition of acrylic acid-modified AuNPs (LOD = 1 µM) [58]. The aggregation of AuNPs occurs through cascade reactions: glucose oxidase (GOx) enzyme produces H_2_O_2,_ which, in the presence of Fe^2+^, leads to AuNPs aggregation, with consequent visual color change. This is one of the first reported examples presenting limited visual color distinction compared to other approaches involving anisotropic plasmonic NPs.

**Figure 2:**
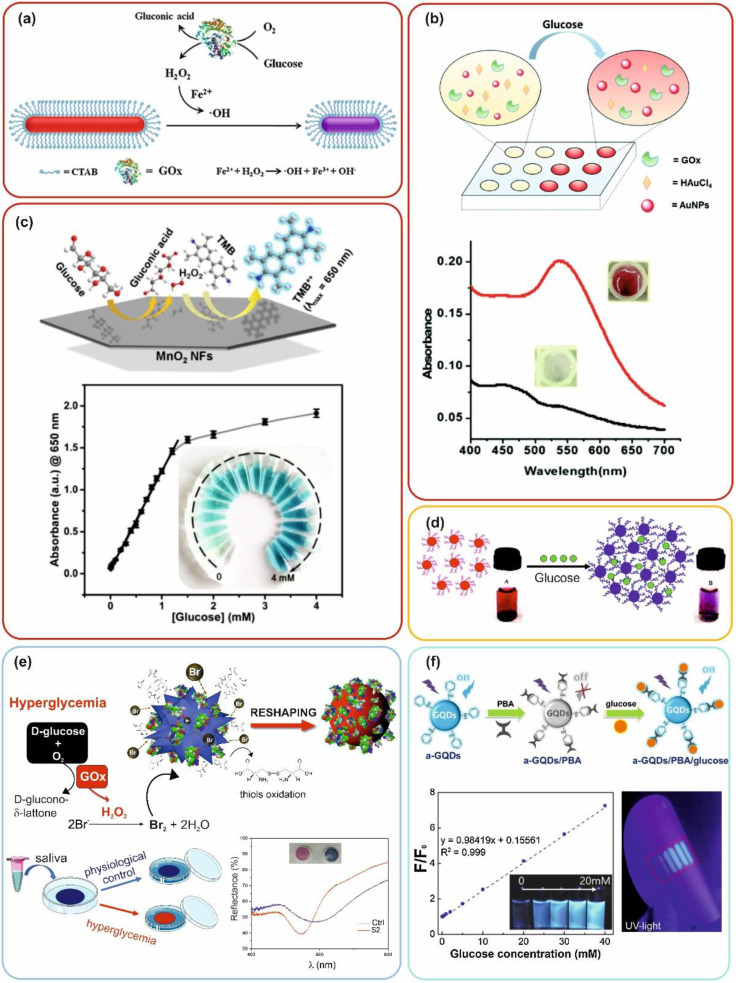
Examples of naked-eye nanosensing strategies for glucose detection including (**a**) etching (Reprinted with permission from Ref. [60]; Copyright 2021 Royal Society of Chemistry), (**b**) growing (Reprinted with permission from Ref. [66]; Copyright 2021 Royal Society of Chemistry), (**c**) nanozymes-chromogenic substrates (Reprinted with permission from Ref. [67]; Copyright 2021 John Wiley and Sons), (**d**) aggregation (Reprinted with permission from Ref. [82]; Copyright 2011 Analytical Chemistry), (**e**) reshaping [83], and (**f**) luminescent enhancement (Reprinted with permission from Ref. [84]; Copyright 2021 Elsevier). The color of the frames refers to the biological source used for the assay: (**a**–**c**) = blood, (**d**) = urine, (**e**) = saliva, (**f**) = tears. The Figure’s panels were adapted from the originals.

Rod-like NPs (mainly Au nanorods, AuNRs) have been reported as useful nanotools to develop colorimetric sensors based on the “etching approach”, often involving GOx enzyme [59]. GOx can react specifically with glucose in complex media producing H_2_O_2_ that, via Fenton or Fenton-like reactions, transforms in the free radical and rapidly oxidize the AuNRs; a few examples demonstrated this approach to also be effective in serum (see Figure 2a) [60]. AuNRs present an LSPR band that is very sensitive to little changes in the aspect ratio and, for this reason, they are very promising for semi-quantitative naked-eye detection. During the etching process, multiple colloidal suspension colors (from green to blue, violet, and red) can be obtained depending on glucose concentration.

A similar sensor for serum glucose determination using AuNR etching was developed involving enzymatic tandem reactions and halogen ions. Specifically, the glucose oxidation by GOx leads to H_2_O_2_ formation, which reacts with HRP generating hydroxyl radicals ·OH, which promotes oxidative etching [61]. Halogen ions (i.e., Br^−^, I^−^) were employed to enhance the etching. The serum samples were diluted 50 times for testing. This mechanism allowed to reach faster reactions when compared to the use of the Fenton reagent. Since the in vitro assay requires multiple additions, all reagents were embedded in a silica gel inside an Eppendorf tube to simplify the biosensor use. In this way, the sample can be added directly to the gel, but the slow diffusion of the glucose through the gel entails some limitations. Furthermore, a relatively high concentration of HRP is needed in this system, which can limit the reaction conditions.

HRP can be employed to oxidize chromogenic substrates quickly, and this system can be integrated with a plasmonic platform leading to richer multi-chromatic sensing that can improve visual detection. For example, TMB reaction can be coupled with GOx and HRP for AuNR etching to improve the naked-eye color change recognition [62]. The TMB oxidation itself leads to color change, and the AuNR etching by TMB^2+^ results in another color change. This strategy allows for building a more extensive range of different colors obtained by variation of glucose concentration.

An in vitro colorimetric assay based on the etching of Au nanobipyramids instead of AuNR was employed to detect glucose in serum samples (diluted 100 times) [63]. Furthermore, in this case, two enzymes are involved in the detection process: GOx and HRP. The hydroxyl radicals generated promoted oxidative etching of the nanobipyramids and consequent LSPR shift and color change of the colloidal suspension. The use of enzymes leads to good selectivity of the biosensor, and the anisotropic shape allows for multiple color changes in the glucose concentration range of interest (0.05–90 mM with a detection limit of 0.02 mM). In this example, the etching of bipyramid geometry was shown to be more efficient than the rod’s one.

There are disadvantages related to the use of HRP, which is quite expensive, presents poor long-term stability in solution [64], and its activity is quite sensitive to the biological environment; thus, it is maybe inconvenient when aiming at a cost-effective commercial product. Furthermore, the use of two enzymes inevitably leads to some compromise in their efficiency due to the difficulty of finding a unique working condition (such as temperature, pH, buffer solution, ionic strength) optimal for both of them.

Several HRP-free systems were indeed reported. Morphological transformation of prism-like AgNPs to disk-like shape by GOx-produced H_2_O_2_ was developed, obtaining high sensitivity (LOD = 0.2 µM) [65]. However, the test required 40 min, and the serum samples needed pre-treatment and 100-time dilution to avoid matrix effect (interference).

Alternatively to the etching, growth strategies were also adopted to develop a serum glucose assay (LOD = 49 µM) [66]. The detection of glucose can be based, for example, on seed-mediated growth of AuNPs where the GOx-produced H_2_O_2_ acts as a reducing agent of the gold precursor in solution depositing on 5 nm AuNPs, increasing their size and therefore their optical density (OD), see Figure 2b. A suspension having 5 nm AuNPs at relatively low concentration (i.e., <10 nM) is colorless, while the same concentration of larger AuNPs (>30 nm) is typically red (at least when the NPs are spherical). Measurements were optimized for a 30 min test, and serum samples were diluted 10 times for testing. In this condition, no significant interference by the serum biomolecules was observed, including potential reducing agents (i.e., ascorbic acid) due to their low concentration [66]. Clinical samples were also analyzed, validating the potential of this strategy for diagnostic purposes. While this approach can be considered interesting for in-lab high-throughput screening, its translation to a home-test still requires significant implementation. The gold salts solution typically employed (i.e., HAuCl_4_) presents poor long-term stability, and the test was optimized only in vitro (in solution) and only for serum samples (not whole blood), which also required dilution (1:10). Moreover, as previously discussed, approaches based only on changes of color intensity might limit naked-eye distinction and, therefore, might be less competitive in terms of potential applications for semi-quantitative measurements, compared to color change strategies unless employing a smartphone app.

An interesting approach is to replace natural enzymes with nanozymes. Metallic nanozymes typically exhibit peroxidase-like activity that can be exploited in colorimetric sensors using chromogenic substrates [45], but they commonly lack GOx-like activity. Interestingly, MnO_2_ two-dimensional nanoflakes were reported to simultaneously act as GOx and peroxidase, allowing for one-pot colorimetric strategies (see Figure 2c) [67]. Furthermore, the dual enzymatic action of MnO_2_ nanoflakes induces a cascade reaction. The glucose reacts with the nanozyme, producing H_2_O_2_ that directly transforms into hydroxyl radicals in situ, eliminating the mass transfer process of reactants and intermediates, thus accelerating the reaction rate and the colorimetric outcome. The test was carried out at 37 °C for 15 min (LOD = 1 µM). This strategy was successfully tested in full serum. In this example, a limit might be related to the intensity-dependent TMB readout (challenging to be semi-quantitative by naked-eye). Moreover, the nanozymes might not be as selective as GOx, and the operational conditions were set above room temperature, which can be a limit for home-testing purposes.

Similarly, direct glucose detection in whole blood by a colorimetric assay based on GOx-conjugated graphene oxide/MnO_2_ nanozymes was performed both in vitro and using a plasma separation pad. In this work, blood samples were analyzed without pre-treatments or dilutions. Linking the GOx to the nanozyme (acting as peroxidase), the system performed an enzymatic tandem reaction in one single step. The consequent reaction with a chromogenic substrate (TMB) led to visual detection of glucose in a few minutes (LOD = 0.17 mM) [68].

A paper-based analytical device using a GOx-loaded pluronic-based nanocarrier functionalized with an artificial peroxidase was recently developed and tested in human serum samples (diluted 10 times). The one-pot enzymatic cascade reaction leads to oxidation of a chromogenic substrate (2,2′-azino-bis(3-ethylbenzothiazoline-6-sulfonate). The color change obtained on the pad in the presence of increasing glucose concentrations was measured with a smartphone app [69].

Few studies report glucose sensors based on hollow Au-Ag alloys, mainly tested in diluted serum [70,71,72]. The strategy is commonly based on the selective/preferential dissolution of Ag by the GOx-produced H_2_O_2_, which leads to LSPR shift and color change. In one example, the Au-Ag hollow-based nanosensors were tested in 1:50 diluted serum, whole blood, and urine. While performing well on diluted serum and urine, problems were reported when working in the blood, due to the interference of catalase decomposing H_2_O_2_ to H_2_O and O_2_ [72].

A few more examples shown to be effective both in serum and urine will be discussed in the following section since, as previously mentioned, the serum is less suitable than urine for home-testing translation [72,73].

### 4.2. Urine Glucose Nanosensors

Albeit blood glucose monitoring is more common, testing urine samples is useful to verify possible kidney dysfunctions. Glycosuria is mainly due to untreated diabetes and, in rare cases, to a syndrome where urine hyperglycemia corresponds to hematic glycemia within physiological values [74,75]. Nevertheless, the presence of anomalous glucose concentration in urine is mainly related to diabetes. Since urine is collected in the bladder, where it stays for a certain amount of time before being excreted, the glucose concentration measured is an average of the glycemia over that amount of time. Therefore, the shorter the collection intervals, the more accurate the measurement and the correlation with blood glucose variation will be.

Commercial blood glucometers are not suitable for urine, and GOx-peroxidase (involving an H_2_O_2_ sensitive molecular chromophore) and hexokinase methods are often used as standard laboratory approaches. However, as previously mentioned, peroxidase (e.g., HRP) presents some disadvantages, including weak stability, small-scale production (challenging to scale up), and sensitivity to the pH and temperature (it denaturates easily). Some alternative methods have been reported for urine glucose detection based on electrochemical sensors [76,77], electronic nose [78], molecular-based colorimetric [79], and luminesce-based sensors [80,81]. Here, we only focus on colorimetric nanosensors with the potential for home-testing translation.

Naked-eye detection of glucose in urine was performed by aggregation strategy employing AuNPs functionalized with GOx (Figure 2d) [82]. Interestingly, the GOx bioconjugation on the AuNP also enhances the enzyme stability and resistance to degradation. When glucose exceeds 100 μg/mL of concentration, the red suspension turns blue. The reaction is instantaneous, with no need for incubation time. To simulate the urine composition of a diabetic subject, a urine sample from a healthy subject was altered by glucose spikes, and albumin/creatinine (ratio 40 mg/mM) was also added to mimic diabetic nephropathy. With this approach, rather than quantitative or semi-quantitative measurements, efficient screening for a critical pathological glucose concentration can be provided.

An in situ AuNR etching strategy was developed based on the following mechanism: GOx generates H_2_O_2,_ that in the presence of MoO_4_^2−^ rapidly oxidizes the iodide ions (I^−^ to I_2_), which in turn act as a potent etching agents [85]. The molybdate catalyst significantly accelerates the I_2_ production overcoming the relatively low etching rate often due to the presence of CTAB, commonly employed as the stabilizing agent in AuNR preparation. AuNR corrosion mainly occurs along the longitudinal direction (presenting a smaller density of passivating ligands and higher surface energy/reactivity), leading to LSPR blue-shift and color change (LOD = 0.1–0.3 μM by naked-eye) [85]. The relatively high temperature employed (45 °C) in addition to the relatively long test time (25 min) and the necessity for a 1:500 urine dilution might limit a quick translation of this approach to a home-testing assay.

Recently, a similar etching strategy, but avoiding the use of the catalyst and therefore mainly based on the iodine activity, was also applied to Au nanobipyramids, obtaining visual glucose detection in urine (LOD = 0.35 μM) [86]. Due to the sharper edges of this shape compared to AuNRs, the LSPR appears to be more sensitive to changes in the chemical environment and to the etching reaction. However, the reported test time was quite long (60 min at 37 °C).

A phosphorescence paper-based sensor involving bimetallic (Au-Ag) nanoprisms capped with metallorganic framework (MOF) was reported [73]. The Ir–Zn_e_ MOFs act as luminophore units in virtue of their well-known oxygen-sensing properties. Glucose-GOx reaction is accompanied by oxygen consumption, increasing the intensity of the phosphorescence emission. The plasmonic nanoprisms act as a light antenna and enhancer of the emission intensity, improving the detection limit of glucose sensing. The LSPR of nanoprisms is more intense than the corresponding spherical one, and the wavelength is more suitable for coupling with the MOFs dipole upon light absorption, which is behind the phosphorescence enhancement. The light emission of the assay is visible by naked-eye; however, the measurements performed with this assay relied on spectroscopy. The urine samples were protein depleted and diluted 100 times before analysis to avoid biomolecule interference.

ZnFe_2_O_4_ magnetic NPs are nanozymes with peroxidase-like activity and, coupled with GOx and the chromogenic substrate TMB, were employed to develop a colorimetric sensor for urine glucose [87]. The test required at least 40 min and a temperature of 37–40 °C. Therefore, significant implementation would be needed for practical home-testing.

A similar approach (Glucose-GOx-TMB) was developed by using maghemite (y-Fe_2_O_3_) cubic NPs as peroxidase-like nanozyme (LOD = 0.2 μM) [88]. The system was tested using diluted urine samples. Despite the potential for naked-eye detection, these reports only focus on spectroscopic analysis for glucose concentration determination.

The same strategy was also applied using Graphene Oxide-Fe_3_O_4_ magnetic hybrid nanocomposites [89]. Both of them separately mimic peroxidase and, when together, they enhance the catalytic activity. Furthermore, the magnetic properties can be exploited to remove the dark NPs with a magnet to make visual detection easier. Urine samples were centrifuged for 40 min, and then the supernatant was diluted 10 times prior to analysis.

A free-standing nanozyme sensor was realized embedding AgNPs within a 3D cotton fabric matrix [90]. While in most cases, the nanozymes (and NPs in general) are employed as a colloidal suspension and can be accompanied by problems involving uncontrolled aggregation in biological media (elicited by the high ionic strength and biomolecular corona formation) [91,92,93], in this study, the NPs were immobilized on a substrate. Therefore, the cotton fabric acts as a template allowing for a high number of catalytically active sites and absorbent properties, which also helps in the rapid adsorption of glucose during the urine assessment. The colorimetric readout was provided by the NPs-generated hydroxyl radicals oxidizing a chromogenic substrate (i.e., TMB, OPD, ABTS), with a visual LOD = 0.1 mM. The urine samples testing presented no significant interference by the biological environment; however, urine samples were diluted when needed. Interestingly, the device was reusable up to 10 times. Conceptually this example probably represents the closer approach to a home-testing prototype for urine glucose detection. Visual colorimetric calibration or a smartphone app for color measurements would allow for a semi-quantitative instrument-free assay. However, a clinical trial is needed to validate the test accuracy on a larger number of real samples (only samples from two donors were tested). Considering the donor-dependent urine composition variability, the interference and accuracy should be carefully re-assessed.

### 4.3. Saliva Glucose Nanosensors

Saliva is mainly a mixture of parotid secretion (20–25%) and submandibular secretion (70–75%), with a small contribution from sublingual, tubarial, and minor glands. Main constituents include water (98%), mucopolysaccharides and glycoproteins, electrolytes, white blood cells, epithelial cells, proteins, and enzymes [94,95]. The daily production of saliva ranges from 0.5 to 1.5 L; the standard flow rate is about 0.3 mL/min, increasing up to 7 mL/min when stimulated [96]. Saliva is a readily available biomarker source with a straightforward non-invasive collection; moreover, it is colorless, a clear advantage for colorimetric tests. However, saliva sampling must be performed with rigor to avoid misleading results. Saliva samples can be divided into stimulated and non-stimulated. While in some cases it may not be crucial, this aspect is particularly relevant for glucose measurements. To better correlate with the hematic values (and potential risks of diabetes) and improve reproducibility, only non-stimulated saliva should be employed [21,97]. Furthermore, salivary glucose presents a low concentration compared to the blood one (see Figure 1), thus requiring highly sensitive methods.

Electrochemical sensors with good potential for salivary glucose detection have been published [98,99,100]. Still, these approaches require instrumentations, often need calibration procedures, and present relatively high production costs, especially if compared to rapid colorimetric kits. Due to the low salivary glucose concentration, the development of efficient naked-eye tests is undoubtedly very challenging. Nevertheless, a few interesting examples have been reported [101,102], including a small number of promising nanosensors for future home-testing applications, which are discussed in this section.

Silica NPs coated with organosilanes (3-[2-(2-amino ethylamino) ethylamino] propyl trimethoxy silane (AEPTMS) and octyl trimethoxy silane (OTMS) were employed to coat a glass plate, obtaining a pH-responsive substrate for glucose detection. Depending on the pH, the prepared substrate can switch from superhydrophilic to superhydrophobic in virtue of protonation/deprotonation of the NPs functional group responsible for the wettability [103].

A drop of the sample was deposited on the assay’s substrate after incubation with GOx. When the glucose concentration increases, the GOx produces more gluconic acid, decreasing the pH, hence decreasing the drop’s contact angle and resulting in a visible change of the drop shape (flat in case of hyperglycemia, round for physiological conditions) with LOD = 0.32 nM. This original visual detection assay presents the advantage of being suitable for color-blind subjects. The method was validated on saliva (urine and sweat) samples from nine healthy donors and nine diabetic subjects. Nevertheless, the prolonged assay time (60 min) might represent a limit for POC applicability [103].

As seen for the other biofluids, a relatively reliable colorimetric approach is based on artificial nanozymes combined with GOx and TMB chromogenic catalyzed oxidation reaction. In a recent work, polyethyleneimine-stabilized PtNPs presenting notable peroxidase-like activity were employed for salivary glucose detection (LOD = 0.15 mM) [104]. Polyethyleneimine provides enhanced stability in the biofluid, keeping most of PtNP catalytic surface available and allowing detection in saliva samples. However, in this latter case, the reported assay time was also quite long (60 min).

A similar colorimetric strategy was employed exploiting nanoporous MOF nanozymes oxidizing TMB [105]. The MOF comprises a 4-amino-3-hydrazino-5-mercapto-1,2,4-triazole (AHMT) as a linker, coordinating Pd(II). The nanosensor was embedded in an agarose gel together with the GOx as a portable kit. This assay was validated on saliva (and tears) samples, which required a quite complex pre-treatment involving the protein content removal (boiling and centrifuging steps) not suitable for home-testing applications.

Another GOx-nanozyme-TMB sensing approach was developed by using MoS_2_ quantum dots decorated AuNPs [106]. MoS_2_-QDs surface sulfur atoms allow for strong Au-S bond formation, increasing the stability of AuNPs and promoting the charge transfer, enhancing the catalytic activity. All reagents were embedded in agarose gel as a portable kit. The colorimetric assay was validated for glucose detection on pre-treated (protein removal) and diluted saliva (tears and serum) samples. The reported assay time was 40 min.

Recently, a paper-based device involving plasmonic AuNPs was shown to be effective on whole saliva samples. Unlike the previously discussed etching-based approaches, accompanied by loss of color intensity due to significant nanostructure corrosion needed to obtain naked-eye appreciable color change, this example relies on an optical density conservative reshaping process [83].

Au spiky nanostructures [107], in the presence of GOx-produced H_2_O_2_ and halogen ions (i.e., Br^−^ or I^−^), undergo rapid structural reorganization, leading to nanospheres, passing from blue to red color in 10–15 min (see Figure 2e). Since the AuNP surface does not present strongly bonded ligands nor surfactants (such as CTAB) that, as previously discussed, commonly slow down the etching processes, there was no need for a catalyst to obtain a rapid color change. Interestingly, when using saliva samples, the protein corona formation, instead of leading to colloidal stability-related problems, seemed to stabilize the AuNPs, leading to a more controlled and reproducible reaction mechanism. A home-testing kit prototype was developed by depositing/immobilizing the reagents on a nylon micro-pad. The assay was validated by a small clinical trial involving 20 subjects (including both diabetic and healthy donors) using non-stimulated saliva with no purification steps. The visual LOD calculated by testing whole saliva samples was 0.02 mM. Despite the potential for smartphone-assisted semi-quantitative measurements, the assay was optimized for ON/OFF outcomes and, therefore, mainly applicable for POC screening to discriminate hyperglycemic samples above a critical concentration threshold.

### 4.4. Tears Glucose Nanosensors

Tear glucose monitoring represents another approach for non-invasive diagnosis of diabetes since, compared to blood, it is more accessible and continuously available. However, glucose concentration in tears is small (see Figure 1), sample collection for in vitro testing is quite challenging, and due to the little volumes, it can be time-consuming [108]. It is therefore challenging to make tear glucose portable devices competitive with current commercial blood glucose assays. Several articles refer to onion-induced tearing for sampling [109], which is quite discomforting if not invasive. A smart alternative is developing wearable devices for continuous monitoring [28,29,110]. Tear glucose sensors involving promising in situ polarimetric approaches [111,112], in vitro colorimetric platforms [113] (mainly involving organic chromogens), and chemiluminescent assays [114] have been reported. However, there is still considerable room for improvements, and here we discuss a few interesting examples of nanosensors applied in this context.

Two examples involving colorimetric nanozyme-based strategies validated in tears and saliva samples were reported in the previous section since they perform better in saliva; onion-induced tearing sampling was employed for in vitro testing [105,106].

AuNPs functionalized with the fluorophore Rhodamine B isothiocyanate (RBITC) were investigated as a fluorescent sensor. Firstly, AuNPs were synthesized with activated dextran as a reducing and stabilizing agent and conjugated with aminophenyl boronic acid (ABA), forming a Schiff base. Then, the fluorophore was chemisorbed on AuNPs surface via hydrogen bonding. RBITC presents intense fluorescence properties in the visible range but is quenched by AuNP proximity. Glucose can form covalent bonds with boronic acid, displacing the fluorophore that, once free, can emit the light signal. This strategy was applied on real tear samples of five female volunteers with the onion-induced method. However, despite the fluorescence in the visible range and the potential for visual/smartphone detection, the naked-eye detection was not investigated in this study [115].

On the contrary, a similar strategy exploiting graphene quantum dots (GQDs) was shown to give a visually appreciable response [84]. GQDs are biocompatible, non-toxic (compared to semiconductor QDs), and present good photoluminescence stability. Aniline functionalized GQDs, once exposed to phenyl boronic acid (PBA), are quenched by the electrostatic interactions. However, when glucose is in solution, PBA disassembles from the GQDs surface to form boronic ester complexes with glucose, and GQDs emit the light signal (see Figure 2f). The increasing concentration of glucose leads to a blue light brightening. The system was printed on filter paper for a portable application, and the paper-based glucose sensing was performed on diluted artificial tears showing good potential. Unfortunately, no validation was performed on real biological samples.

Nickel and nitrogen-doped graphene nanotubes (GNTs) decorated with Pt-nanozymes were employed for visual tears (and saliva) glucose detection. While nickel is supposed to improve the catalytic activity of PtNPs, nitrogen-doping was done to improve and promote the PtNP assembly with the nanotubes. Therefore, the PtNPs on the nanotube surface gained stability and enhanced the peroxidase catalytic activity [116]. GOx was employed to produce H_2_O_2_ from glucose. The visual detection was achieved via nanozyme-mediated catalytic oxidation of TMB, leading to a blue color solution with a glucose concentration-dependent intensity. In vitro testing was performed on real tear samples containing glucose spikes by the addition of the nanohybrid system solution (plus GOx and TMB), and the color change was obtained in 5 min. The assay was also tested in saliva but was better performing in tears. A translation of the colorimetric systems on a paper-based microfluidic device was also done, depositing all the reagents in line in physically separated spaces on the substrate. However, the paper-based system was only tested in buffer solution.

### 4.5. Sweat Glucose Nanosensors

To perform glucose measurements in sweat, it is essential to employ wearable devices, like skin patches. Among the sweat glucose detection strategies, NPs-based electrochemical wearable sensors are the most explored [117]; however, a few interesting colorimetric approaches with potential for instrument-free applications have also been reported.

One previously mentioned example of naked-eye nanosensor performing in saliva and sweat involving a silica NPs-based pH-sensitive substrate [103] reacting to GOx-produced gluconic acid has already been discussed in the *Saliva Glucose Nanosensors* section. This device was validated with real sweat samples from nine healthy donors and nine diabetic subjects. However, the long assay time (60 min) could limit the sensor applicability.

The detection of glucose in sweat was also achieved using bioinspired fluorescent nanodots (bidots) non covalently conjugated with glucose oxidase [118]. The H_2_O_2_, resulting from the GOx oxidation of glucose, in the presence of Fe^2+^ (by Fenton reaction) transforms into hydroxyl radicals that quench the bidots fluorescence. However, the fluorescence quenching related to increasing glucose concentration was only estimated by spectroscopy, and the assay only tested on artificial sweat.

Real sweat samples analysis was achieved with a wearable skin pad involving hybrid nanocomposites made of porous silicon nanoparticles (PSi), plasmonic nanoparticles, and CQDs [119]. The red-emitting PSi were loaded with blue-emitting CQDs and assembled with AuNPs and AgNPs. The plasmonic nanoparticles, in this context, strongly enhance the luminescence of PSi thanks to the interaction between emission dipoles and LSPR. The nanohybrids display red fluorescence since CQDs are completely quenched by PSi. When the sensor is incubated with glucose solution and GOx, the produced H_2_O_2_ leads to the oxidation of PSi. The fast release of Si, further enhanced by the bimetallic NPs that increases the electron transition efficiency, allows for a change in the fluorescence from red to blue, shown to be easily distinguishable by naked-eye (upon UV excitation, in dark conditions). The wearable skin pad fabrication was achieved by transferring the system on a transparent and biocompatible chitosan film. This system was tested in vivo on the back area of the neck of some volunteers before and after sleeping. The strategy presents limitations in clearly distinguish samples by naked-eyes because of the low difference in the amount of glucose in sweat. Nevertheless, the detection was achieved with a smartphone presenting UV integrated light source.

In Table 1 are summarized the instrument-free methods, discussed in this Review, tested in the different biological fluids.

## 5. Conclusions and Perspectives

This work presented an overview of the main instrument-free NPs-based strategies employed to date for glucose detection in biological fluids and their potential (and limits) for developing cost-effective rapid POC devices.

The advantages and drawbacks of non-invasive biofluids in this context were taken into account, including the impact of their biomolecular interferent, the different glucose concentrations, and the suitability for home-testing.

In most cases, we believe that the real potential of the described colorimetric strategies as portable sensors would probably be enhanced relying on their translation from in-solution in vitro tests, often requiring multiple steps, to single-step paper-based devices. The examples based on such an approach were the most promising. Since most of the reported methods were designed for blood analysis but tested in diluted (1:10–1:100) serum to avoid interference, it would be interesting to adapt them and test their potential applicability in other non-invasive biological media and/or implement them with LFDs (exploiting rapid in-situ membrane-based filtration).

The unique physical-chemical properties of NPs can allow overcoming the difficulties related to the detection of glucose content in non-invasive fluids, especially if aiming at rapid screening of hyperglycemia involving an ON-OFF approach for a set range of critical glucose concentrations. However, to the best of our knowledge, there are currently no instrument-free methods tested by statistically relevant clinical trials, able to perform quantitatively. Nevertheless, etching/reshaping of anisotropic plasmonic nanoparticles (presenting concentration-dependent multiple color change) and the nanozyme-colorimetric substrate systems held good promises for semi-quantitative naked-eye home-testing devices, even if the second one should probably rely on smartphone-assisted measurements (since mainly accompanied to color intensity changes).

In conclusion, while we are not yet ready to get rid of instruments for glucose monitoring, we believe that shortly, with some further effort in this context, there will be several concrete possibilities for non-invasive tests based on colorimetric nanosensors, which could significantly impact and improve people’s life quality and healthcare costs.

## Figures and Tables

**Table 1:** Summary of the Nanomaterials and approaches described in this Review (example in artificial biofluids are not included).

Nanomaterials	Approach	LOD * (mM)	Advantages **	Disadvantages **	BF ***
AuNPs [58]	Aggregation	1 × 10^−3^	one of the first examples	Slow, limited color distinction	SE
AuNPs [82]	Aggregation	2.8 × 10^−2^	Rapid, color change	ON/OFF system	U
AuNPs [66]	Growth	4.9 × 10^−2^	Signal increase	Slow, sample pretreatment, single color	SE
AuNRs [60,61,62]	Etching	1 × 10^−2^/1 × 10^−1^	Rapid, multicolor, visually semiquantitative	Sample pretreatment, Signal decrease	SE
AuNRs [85]	Etching	1 × 10^−4^ (A)/3 × 10^−3^ (V)	Multicolor	Slow, sample pretreatment, Signal decrease	U
Au Nanobipyramids [63,86]	Etching	2 × 10^−2^/3.5 × 10^−4^	Multicolor, visually semiquantitative	Slow, sample pretreatment, Signal decrease	SE
Multi branched AuNPs [83]	Reshaping	2.2 × 10^−2^ (A-V)	Rapid, multicolor, no signal decrease	ON/OFF system	SA
Au-Ag NPs [70,71,72]	Etching	-	Color change	Slow, sample pretreatment	SE, U
Au-Ag nanoprisms MOF capped [73]	Phosphorescence	3.8 × 10^−2^	Rapid	Sample pretreatment, single color	U, SE
AgNPs [90]	Nanozyme	8 × 10^−2^ 0.1 (V)	Free standing reusable sensor, semiquantitative	Slow, sample pretreatment, single color	U
Ag Nanoprism [65]	Etching	2 × 10^−4^	Multicolor	Slow, sample pretreatment	SE
PtNPs [104]	Nanozyme	1.5 × 10^−1^	No sample pretreatment	Slow, single color	SA
SiO_2_ NPs on a glass substrate [103]	Drop contact angle switch	3.2 × 10^−7^	Suitable for color blinds	Slow, sample pretreatment	SA, U, SW
ZnFe_2_O_4_ NPs [87]	Nanozyme	3 × 10^−4^	Magnetic NPs removal	Slow, sample pretreatment, single color	U
y-Fe_2_O_3_ NPs [88]	Nanozyme	2 × 10^−4^	Magnetic NPs removal	Sample pretratment	U
Graphene oxide/MnO_2_ nanocomposites [68]	Nanozyme	1.7 × 10^−1^	Rapid, no sample pretreatment	Single color	RB
Graphene oxide–Fe_3_O_4_ nanocomposites [89]	Nanozyme	7.4 × 10^−4^ 5 × 10^−3^ (V)	Magnetic NPs removal	Slow, single color	U
GNTs-PtNPs [116]	Nanozyme	1 × 10^−9^	Rapid	Single color	T
Nanoporous polymeric MOF [105]	Nanozyme	4.7 × 10^−5^	Stable	Slow, sample pretreatment, single color	SA, T
MnO_2_ nanoflakes [67]	Nanozyme	1 × 10^−3^	Rapid, emzyme-free, semiquantitative	Single color, reduced specificity	SE
Mn_2_BPMP-PNC [69]	Nanozyme	2.2 × 10^−2^	Rapid, one step	Single color, sample pretreatment	SE
MoS_2_ QDs decorated AuNPs [106]	Fluorescence	6.8 × 10^−5^	Stable	Slow, sample pretreatment, single color	SA
Hybrid NPs (SiO_2_/Au/CQDs) [119]	Fluorescence	--	Continuous monitoring	Single color	SW

* LOD: analytical LOD; V: visual LOD. ** Rapid: ≤20 min; Slow: >20 min. *** BF: biofluid; SE: serum; RB: rat blood; U: urine; SA: saliva; T: tears; SW: sweat.

## Data Availability

Not applicable.
